# Supplementary material for: Are carbon emissions trading and green financial instruments synergistic? -Comprehensive quantitative research based on content analysis
Source: PLoS One. 2024 Mar 7;19(3):e0298601. doi: 10.1371/journal.pone.0298601 (PMC10919601; doi:10.1371/journal.pone.0298601)
Supplement: S1 Data — (ZIP) [file pone.0298601.s001.zip › Data/地级市Z_F工作报告.zip!/╜¡╦╒/╦▐╟¿╩╨/└┤╫╘╣1⁄2╓┌║┼í╛┬φ┐╦╩2╛▌═°í┐_2018.docx]

各位代表：

现在，我代表市人民政府向大会作工作报告，请予审议，并请市政协各位委员和其他列席同志提出意见。

2017年政府工作回顾

过去的一年，我们在省委、省政府和市委的坚强领导下，在市人大、市政协监督支持下，认真贯彻落实党的十八大、十九大精神，始终以习近平新时代中国特色社会主义思想为指导，坚持稳中求进工作总基调，围绕建设“强富美高”全面小康新宿迁，践行新发展理念，扎实推进供给侧结构性改革和“两聚一高”新实践，认真落实“三项任务”，推动“三转三稳”，统筹做好改革发展稳定各项工作，经济社会保持平稳健康发展，新一届政府工作实现良好开局。预计全年实现地区生产总值2600亿元、增长7.3%左右；一般公共预算收入200.6亿元；全体居民人均可支配收入增长9.3%。以全国第一通过国家卫生城市复审，以年度测评全国第一、三年综合评分全国第一的优异成绩摘得全国文明城市桂冠。

一是认真贯彻落实党的十九大精神，全面小康建设迈出坚实步伐。把学习好、宣传好、贯彻好十九大精神作为首要政治任务，专题召开市政府党组（扩大）会、常务会集中学习，深入县区、企业、社区宣讲，坚持原原本本学、原汁原味学，领会精神实质，把握深刻内涵，推动全市各级政府及其组成部门，切实把思想和行动统一到十九大精神上来，把力量凝聚到十九大确定的各项目标任务上来，自觉用习近平新时代中国特色社会主义思想武装头脑、指导实践、推动工作。坚持把学习贯彻十九大精神与落实省委十三届三次全会、市委五届五次全会要求相结合，与宿迁发展实际相结合，与工作谋划、工作推进相结合，有力推动了十九大精神落地生根。以十九大精神为指引系统谋划新路。认真落实新发展理念和“两山”理论，积极探索“生态优先、绿色发展”新路径，系统谋划生态经济示范区和“江苏生态大公园”建设，编制生态经济示范区《发展规划》和《实施意见》，配套出台22个专项文件，推进生态经济示范区建设“十大示范工程”，实施“生态+”“互联网+”“旅游+”三项行动计划，开展8个生态经济示范镇和大运河、古黄河“两河”生态产业富民示范廊道建设，大力发展绿色生产力，加快转变发展方式、优化经济结构、转换增长动力。以十九大精神为指导稳定经济运行。针对经济形势的变化，坚持超前研判、科学施策、精准发力，适应把握引领新常态，统筹推进供给侧结构性改革，积极采取清费减负、强化要素保障、优化营商环境、扩大有效投入等举措，狠抓以制造业为重点的实体经济发展，有效顶住经济下行压力，主要指标增长保持在合理区间，经济运行呈现出“稳中有进、进中向好、好中出新”的良好态势。以十九大精神为动力扎实推进全面小康建设。围绕决胜全面建成小康社会的各项任务，聚焦经济社会发展突出问题，积极回应广大群众民生诉求，千方百计抓重点、补短板、强弱项，统筹做好产业升级、城乡发展、深化改革、生态建设、民生保障等各项工作，全面小康社会建设取得新的进展。

二是全力推动产业转型升级，经济发展质态明显优化。坚持以质量和效益为中心，聚焦规模集聚、提质升级，产业发展呈现出结构优化、质态提升的良好态势。工业经济扩量提质。产业规模快速扩张。持续加大招商引资力度，成功举办“接轨上海”系列活动、2017绿洽会、中德（宿迁）圆桌会议等，大力开展“百日会百商”活动，全年引进亿元以上工业项目201个。聚焦“4+4”产业，深入实施“123”行动计划，170个新型工业化项目顺利推进，规上工业增加值同口径增长9%、居全省第一，工业化水平进一步提升。四大特色产业、四大新兴产业量质并进，产值分别增长9.3%、10%，其中智能家电产业新引进申花电器、伊莱克斯等12个项目，产业链条日趋完整，被授予“中国家电产业基地”；绿色建材产业产值增长15%，大亚木业、冠牛木业等一批大项目顺利投产，获批江苏省博士后创新实践基地。积极推进8个先进特色产业园建设，激光产业园一年内集聚了总投资超40亿元的26个项目，北斗电子信息产业园引进飞鹰科技无人机、绿芒科技等11个项目，双双获批省级科技产业园。按照“六个转型”要求，推动开发区加快发展，宿迁高新区升格为国家级高新区，6个南北共建园区全部位居省考核前十，苏宿工业园区连续八年综合排名第一。产业结构持续优化。全面落实“三去一降一补”任务，加快淘汰低端落后产能，着力提高供给质量，产业发展呈现“两升两降”鲜明特征：新兴产业产值占工业总产值比重提升2.4个百分点，高技术产业产值增长近40%；单位工业增加值能耗下降12.8%，高耗能行业产值增幅下降5个百分点，产业结构进一步变“轻”变“绿”。实体经济效益提升。深入推进“511”工程，全年完成兼并重组企业75户，实施超千万元技改项目390个、完成技改投资141亿元，新增股改企业72户、挂牌上市企业12户，新增开票销售超2000万元工业企业390户。规上企业利润率达10%，工业企业入库税收增长25%，均居全省第一，企业发展质量和效益进一步增强。农业现代化水平稳步提高。扎实推进新一轮农业结构调整，农业呈现出规模化、产业化、标准化、品牌化发展态势。新增市级以上农业龙头企业33家，建成2000亩左右成方连片高效农业基地32个，带动全市新增生态高效农业面积26.6万亩，设施农业、设施渔业占比分别提升至17.9%、25.8%。制定14个地方农业标准，农业生产方式更加科学。新增“三品”农产品475个、地标农产品1个，泗洪大米、绿康牌洪泽湖大闸蟹荣获第十五届中国国际农产品交易会金奖。新建家庭农场集群及综合服务中心15个，家庭农场总数达7691家、居全省首位。“一村一品一店”标准村占比达68%，7个乡镇纳入省级农业特色小镇培育名录。现代服务业加快转型。旅游产业蓬勃发展，三台山森林公园成功获批国家级森林公园，洪泽湖湿地公园通过国家5A级景区景观质量评审，双沟酒文化旅游区创成国家4A级景区，衲田风情小镇入围首批省级旅游风情小镇创建名单。三台山衲田花海音乐季和亚洲青年微电影展等活动影响力愈来愈大，央视、腾讯网等国内主流媒体关注直播，网络点击覆盖人群超过2000万人次。全年接待国内外游客2220万人次，实现旅游总收入255亿元，分别增长15%、18%。宿豫区被评为全国休闲农业和乡村旅游示范县（区）。电子商务加速崛起，按照“一区多点”总体布局，引导电子商务产业集聚发展，8个省级以上电子商务示范基地新入驻企业260余家、总数超过800家。建设运营地方特色馆10个，高起点规划建设京东·台企名品馆，积极助推“台商走电商”。建成乡镇电商服务站114个，发展农村电商服务点1028个，快递业务量跻身全国50强，全市电子商务交易额达1020亿元、增长37.8%。金融服务水平持续提升，设立信用贷款基金、融资担保风险补偿基金，引导金融机构加大实体经济信贷投放力度，制造业贷款增速位居苏北第一。多措并举推进企业去杠杆，新增直接融资225.8亿元，直接融资占社会融资比重达20.3%。积极创建国家级保险创新综合试验区，保险产业园一年内集聚了6家全国财险10强企业。物流服务体系逐步完善，12个物流重大项目有序推进，京东智慧物流全国运营调度中心投入使用，运河宿迁港开港运营。创新驱动能力不断增强。实施创新型企业培育“百人千企”服务行动，创新建设离岸研发机构，加快技术研发和交易平台建设，省级众创空间、科技产业园数量分别增长133.3%、83.3%，预计科技进步贡献率达53%。机器换人工序普及率、高端装备数控化率分别达51.7%、40.5%，规上企业关键环节互联网应用覆盖率达52%，企业创新能力进一步提升。

三是统筹推进城乡融合发展，城乡建设品质加快提高。着眼构建“133”城镇发展格局，突出“以物应人以物化人、以文育人以文化人”，城乡发展质态和功能内涵发生显著变化。中心城市内涵快速提升。按照“生态、精致、时尚，充满人文关怀”建设定位，着力提高城市营建水平，推进城市建设管理与市民素质同步提高。围绕“以物应人以物化人”，致力通过基础设施的完善、功能配套的优化，满足市民基本需求；通过精品环境的打造、公共服务标准的提升，在潜移默化中改变人的生活习惯，提高文明素养。完善城市功能，提升生活品质。积极推进中央活动区建设，中央商场、仁恒里文创特色街区建成投用，新盛街片区改造项目、西楚活力街区策创营工作扎实推进，银湖广场、电商产业园等城市之家完成建设。深入实施“道路通达”工程，整改断头路、斑马线不通等问题379处，新增公共停车位3800个，建成7个生态立体停车场和便民方舟区域智慧停车系统；新增新能源公交车120辆，万人公交车拥有量超过省平均水平，群众出行越来越便捷。环古黄河绿道工程全线贯通，建成28片小型体育设施，“十分钟健身圈”更加完善。精塑城市形态，美化生活环境。积极推进林荫、彩色、花园、海绵“四个城市”建设，东关口历史文化公园、如意谷黄花槐片区等创意景观建成运营，40个街头绿地和小游园投入使用，一批花廊花墙扮靓城市，基本实现市民出行“300米见绿、500米见园”；大力实施街景提升工程，累计拆除违规户外广告1397处，完成62条道路路灯增亮改造；全面拆除楼顶广告和楼面晾衣架，城市安全隐患大为减少；开展店招店牌改造升级，城市街道更加整洁美观。推进“三场革命”，引领生活方式。全面打响厕所革命、农贸市场革命、垃圾革命，高标准建成“街坊公舍”27座，市民户外休憩有了新场所；按照“农+超”标准，新（改）建菜市场10个；20个市区居民小区、3个乡镇实现垃圾分类收集和处理，市民生活越来越舒心。加强机制创新，提高建设水平。全面推行“333”工作法、具美评审、“EPC”管理模式、导则标准等城市建设管理机制，成功举办“衲田杯”可持续设计国际竞赛，获批全省唯一工程总承包试点市、全国第二批城市设计试点城市，市第一人民医院项目获评中国建设工程“鲁班奖”。围绕“以文育人以文化人”，通过城市文化的熏陶、行为规则的引领，不断提升人的文明自觉和行动自觉。立足陶冶市民情操，开展文汇巷石榴树认养活动，成功放养广场鸽，营造44处落英落叶景观，为城市增添了更多人文气息；28家单位庭院完成开放式改造，群众享受到更多绿色资源、人文空间。立足加深城市记忆，相继命名了石坡岸路、饮马堤路等一批承载城市历史的道路，留住了城市的“根”；紧扣春节、中秋等重要节日，创新打造一批节庆小品，具有宿迁“土味”的人文气息越来越浓。立足文明养成，落实落细“宿迁文明20条”，有序排队不插队、主动让座等文明行为成为市民的自觉习惯；“礼让斑马线·文明我点赞”活动持续开展，试点非机动车推行过斑马线，人脸识别、远光灯抓拍、智能声呐检测等高科技设备广泛应用，文明交通指数继续领跑全省乃至全国，《人民日报》、新华社、中央电视台等主流媒体纷纷报道交通文明的“宿迁经验”。美丽县城建设成效明显。深化市县联动机制，推动市级成功经验向县城延伸，有力提高了县城建设品位和管理水平，成为苏北首个国家园林城市、国家卫生城市县区全覆盖的地区。特色镇村建设扎实开展。按照城市功能、田园综合体和生态涵养三大类布局，实行差别化考核，着力推动乡镇特色发展。推进8个生态经济示范镇建设，初步形成各具特色的发展之路。出台特色乡村建设导则，加快特色田园乡村建设，5个村庄入选省首批特色田园乡村试点。持续开展“三清”行动，实施农村水系沟通、河道清淤整治及改厕工程，乡村环境面貌进一步改善。重大基础设施建设加速推进。徐宿淮盐高铁进展顺利，将于2019年建成通车。宿连航道工程可行性研究报告通过审查，合宿新高铁完成设计招标。宿扬高速宿迁段建成通车，宁宿徐宿迁收费站南移工程完成施工。21项重点输变电工程按期投运，全市电网供电能力提升30%。光网升级改造扎实推进，成为省内首家“全光运营”城市。

四是持续深化重点领域改革，发展内生动力不断增强。以“减权去利便利化”为核心，加大经济体制、农业农村、社会事业等领域改革力度，市场和社会主体活力进一步释放。经济体制改革纵深推进。以“六个一”工程为统揽，深入实施“放管服”改革，基本实现“3550”目标。围绕降低注册准入门槛，开展“多证合一”“全程电子化登记电子档案管理应用”“江苏省开办企业三个工作日完成”3个省级试点，成为全国率先启动网络市场监管与服务示范区创建的地级市，开办企业和不动产登记分别压减到3个、5个工作日以内。围绕优化投资建设流程，扎实推进多评合一、多图联审、区域评审、信用承诺等改革，零用地技术改造项目实行“不再审批”，施工许可和竣工验收均压减到50个工作日以内。围绕释放生产经营活力，工业类产品生产许可制度改革取得新突破，获批省级先证后核改革试点，先证后核类、非先证后核类许可事项办证时间分别压缩到1个、25个工作日以内。持续推进资格资质改革，政府实施的职业资格仅保留29项，资质准入清单由71项压缩至24项，有效破除了群众就业创业束缚。开展全流程清费减负，取消、停征和降低72项行政事业性收费，全年为企业和群众减负约2.3亿元。围绕提升政务服务水平，宿迁政务服务网“四级联动、全网通办”正式启用，“不见面”审批和服务事项达99%以上，群众办事更加方便。探索开展相对集中行政许可权试点改革，成立宿迁经开区行政审批局，审批权限进一步下放。完善社会信用体系，推广政务人员信用管理和“信用+”管理模式，创成国家社会信用体系建设示范城市。农业农村改革深入实施。加快农村土地“三权分置”改革，全面完成农村土地承包经营权确权登记颁证。推进土地增减挂钩改革，探索农村土地承包经营权股份化试点，共成立村级土地股份合作社392家，土地增减挂钩节余指标省域内转让规模和金额均居全省第一。深化“两权”抵押贷款试点，办理贷款数量和金额分别位居全省第一、第二。社会事业改革扎实开展。医卫改革不断深化，政府购买基本公共服务覆盖90%以上的乡镇医院，分级诊疗制度、家庭医生签约服务、医联体建设等稳步实施，在全省率先建立乡村医生待遇增长机制，医卫服务水平进一步提升。运用卫生监督“一平台三系统”，建立医疗机构综合监管、信用管理、第三方评价机制，有力推动医疗机构健康发展。教育改革稳步实施，推进义务教育学校教师“县管校聘”管理体制改革试点，探索教师备案制度管理，全面启动中考中招制度改革，幼儿园独立法人登记工作有序开展。行政执法体制改革加快推进，成立卫生计生、农业、交通运输、文化市场四支综合执法队伍，基本建成简约高效、职责清晰、运转协调、执法有力的市级综合行政执法体系。

五是系统推进生态文明建设，绿色发展优势更加巩固。围绕建设生态经济示范区，全面加强生态保护与治理，生态环境容量不断提升，绿色跨越的基础进一步夯实。“263”行动全面实施。水体治理成效明显，在全省率先通过河长制验收，省考以上断面达标率同比上升3.8个百分点，世纪河等20余条黑臭水体得到有效治理，马陵河整治全面完成，由过去的“龙须沟”蜕变为生态河、景观带。大气治理深入开展，加强对建筑工地、道路、码头等重点区域检查管控，推进高污染燃料禁燃区建设，治理“散乱污”企业，大气质量达到国家“气十条”终期考核要求。空气异味有效控制，加强化工园区企业整治，“三废”排放实现稳定达标，异味扰民问题得到有效解决。土壤治理有序推进，开展设施蔬菜连作障碍防治，积极推进农业标准化生产，化肥、农药施用量持续减少。生态基础更加坚实。污染物收集能力持续提高，新建污水收集管网375.5公里，新铺设尾水压力管道50公里，中心城区雨污分流面积达170平方公里。城乡生活垃圾分类减量和综合治理工作加快推进，沿街店面垃圾实现定时定点收集。污染物处置能力不断加强，启动205个村庄生活污水处理设施建设，新（扩）建城镇污水处理厂61座。推进农业面源污染整治，完成禁养区养殖场关闭搬迁任务。引导农业废弃物收集处置，综合利用率提高到96%、居全省前列。清洁能源供应能力逐步增强，城市热源点和供气管网工程建设全面启动，宿迁至泗洪、沭阳的长输管线完成场站建设，燃气管网建设向开发区和乡镇延伸。碳汇能力进一步提升，启动100万亩生态经济林建设，新建成片林5万余亩；完成杨树更新改造23.96万亩，新增受保护自然湿地9.7万亩。生态保护机制更加健全。率先建立行政执法与司法联动的生态保护机制，完善排污权有偿使用和交易管理体系，实施落后产能常态化淘汰机制和提前主动退出激励机制，单位GDP能耗下降6%左右，化学需氧量、二氧化硫等指标预计完成省下达的减排任务。泗洪县被确定为首批“绿水青山就是金山银山”实践创新基地。

六是千方百计办好民生实事，群众获得感明显增强。始终把人民群众对美好生活的向往作为工作的出发点和落脚点，坚持民生优先的工作导向，持续加大民生投入，民生支出占一般公共预算支出超过80%，民生福祉进一步提升。群众口袋更实了。大力促进富民增收，全体居民人均可支配收入达20716元，增幅居全省第三。城镇累计新增就业5.2万人，开展职业技能培训12.7万人。坚持以创业带动就业，新增全民创业项目3.2万个，扶持近10万人创业；成功举办2017中国·宿迁“互联网+创业”大会、第四届金种子网络创业大赛，全市正常经营网店超过6万家，带动就业40余万人。公共服务更优了。医疗水平有效提升，市公共卫生服务中心投入运行，完成571家村居卫生室提档升级，建立医学检验检查结果互认制度，基本公共卫生服务项目人均经费标准提高20%，医疗卫生服务体系更加完善。教育事业加快发展，中小学BC级校舍加固工程、“八校”建设有序推进，“暖心工程”全面实施，南外仙林分校启动建设，北大附属实验学校顺利完工，新增省优质幼儿园26所、三星级高中2所、四星级高中1所，越来越多的孩子享受到更加公平更有质量的教育。宿迁高师升专列入省“十三五”高等教育布局规划。高考本科达线率73.86%，超过全省本科招生计划率3.71个百分点。公共配套持续改善，完成中心城区70个居民小区二次供水设施改造，惠及群众5.8万户、20余万人。出台“七优”文件，全市商品住宅去化周期降至5.5个月，房屋延期交付等问题得到有效解决，房地产业走上健康发展轨道。整治提升80个老旧小区，改造棚户区2.9万户、货币化安置率达91%，群众居住条件进一步改善。完成农村道路安全生命防护工程818公里，升级改造农村公路480公里，群众出行更加安全、更加方便。文体事业不断繁荣，全年开展文化惠民活动1000余场次，市县乡村四级公共文化设施网络更加完善。文艺精品迭出，5部作品获省“五个一工程”奖，位居全省第四、苏北第一，6部作品获省文华奖；书画摄影作品在省级以上赛展中获奖41次，形成了独特的“书法美术宿迁现象”。全民健身运动广泛开展，中国宿迁生态四项公开赛荣获全国“十佳精品赛事”称号，宿迁籍运动员实现全运会金牌零突破。保障网络更密了。城乡居民医保整合全面完成，在全省率先实现城镇职工、城乡居民异地就医联网结算全覆盖。持续开展社保扩面“幸福行动”，城镇职工“五险”扩面新增33.2万人次。城乡低保标准分别提高到每人每月500元和420元，城乡居民养老保险基础养老金提高到每人每月125元，农村“五保”集中供养和分散供养标准分别提高到每人每年6900元、5900元，城市“三无”老人供养标准提高到每人每年1万元，家庭生活困难留守儿童全部纳入托底保障，城乡特困群体保障水平进一步提升。脱贫达小康的底气更足了。以国家扶贫改革试验区建设为抓手，扎实推进“三大片区”综合开发，积极开展“三进三帮”“挂村包户”活动，大力实施“阳光扶贫”，15万低收入人口、48个省定经济薄弱村顺利脱贫，全面完成年度脱贫攻坚任务。创新“扶贫六项机制”，致力减少贫困代际传递。将产前筛查和治疗纳入医保，对2967名残疾儿童免费实施基本康复服务，兑现大病补充保险补偿金近3800万元、惠及低收入人口1万余人次，资助低收入农户子女就学22万余人次，对困难家庭学生免费实施课外辅导，精准扶贫、精准脱贫成效进一步提升。信访稳定、平安创建、综治工作成效明显，生产安全事故起数、死亡人数同比实现双下降，群众安全感继续排名全省前列。民防、物价、气象、地震、食药品安全、民族、宗教、侨务、人民武装和双拥、档案、党史、地方志等工作实现新进展，妇女、儿童、青少年、老龄、科协、红十字、慈善等事业取得新进步。

七是扎实开展政府自身建设，政务服务水平不断提升。牢记为民服务宗旨，切实转变政府职能，持续改进工作作风，政府执行力和公信力明显提高。施政能力进一步增强。加强对发展大局的研究，密切关注各种具体矛盾，坚持早预见、早研判，稳妥高效处置各类问题和突发事件。妥善处理部分企业因担保和经营不善所带来的系列问题，有效防范了区域性、系统性风险。积极应对重大灾害天气，努力将影响和损失降到最低。持续加大“老大难”问题化解力度，继成功解决耿车废旧物资污染、“大花轿”等重大历史遗留问题后，又相继处置了一批重大难题，骆马湖宿迁水域非法采砂泵船全部完成拆解，洪泽湖周边砂场码头全部取缔，湖区生态大为改善。服务效能持续提升。深化行政审批制度改革，压缩审批事项，优化审批流程，群众办事更加方便。完善公开承诺、首问负责等制度，扎实开展“三服务三促进”，实行重大项目挂钩帮扶机制和重点工作督查会办机制，工作推进更加有力高效。依托12345政府服务热线、“鼎鼎有民”、“星期三政民互动”等平台，从公众的不满意和期待中寻找工作着力点，有效解决群众诉求。12345政府服务热线共接听群众来电28.71万件，按时办结率96.35%，群众满意率95.53%。“网上宿迁”门户网站在全国政府网站绩效评估中位居地级市第三。依法行政深入推进。大力开展“七五”普法，全面落实“谁执法谁普法”责任制。严格执行“三重一大”事项集体决策制度，建立政府重大决策出台前向市人大及其常委会报告制度，完善合法性审查、廉洁性审查和集体讨论决定相结合的行政决策机制，健全新型政府法律顾问制度，强化行政复议规范化建设，施政方式更加科学、规范。邀请市人大、市政协领导及市民列席市政府常务会议，让各界更多了解和参与政府决策。依托“网上宿迁”与“宿迁之声”微博微信、“速新闻”、宿迁手机台，以及《您知道吗》政策解读栏目，全面深化政务公开。自觉接受市人大及其常委会的法律监督和工作监督、市政协及社会各界的民主监督，认真办理市人大代表议案、建议和市政协委员提案，把议案提案办理的过程变成改进和推动工作的过程。自身建设务实有效。牢固树立“四个意识”，坚决维护以习近平同志为核心的党中央权威和集中统一领导，始终在思想上政治上行动上同以习近平同志为核心的党中央保持高度一致。严格落实中央八项规定《实施细则》以及省委、市委《具体办法》，驰而不息改进作风。认真落实党风廉政建设责任制，坚决查纠“四风”，从严控制“三公”经费，加大审计监察力度，大力惩治各类腐败，营造了清正廉洁的政务环境。

各位代表，回顾过去一年的工作，在宏观环境复杂多变、经济社会发展面临各种压力和挑战的形势下，我们积极应对，精准施策，进一步巩固了经济社会发展稳的局面、进的势头、好的状态。一是坚持统筹谋划、科学发展。树立系统化思维，既注重当前的发展，更重视长期的进步；既注重个性问题的处置化解，更注重共性原因的调查分析；既注重某一领域突破，更注重全局整体推进，统筹考量、综合施策，牢牢把握工作主动权。二是坚持尽力尽责、务实担当。始终保持干事创业、奋发有为的精神状态，把责任铭记心中，把使命扛在肩上，从大处着眼、细节发力，一步一个脚印，一点一滴努力，久久为功、善做善成。三是坚持尊重规律、恪守边界。遵从社会发展规律、经济运行规律和市场规律，努力厘清并恪守政府、市场、社会三方边界，充分发挥市场对资源配置的决定性作用，更好地发挥政府作用，不断激发市场和社会主体活力。四是坚持法治思维、依法履职。牢记“法无授权不可为”“法定职责必须为”，自觉用法治思维推动工作开展，用法治手段破解矛盾难题，用法治规范保障发展成果，确保决策和行政不偏航、不越线、不逾矩。五是坚持尊重民意、发展为民。把民意作为最大执政资源，坚持问题和需求导向，从“民声”中找准群众“痛点”“盼点”，确立政府工作“重点”“难点”，认真抓好老百姓最急最忧最怨的事情，切实解决群众最关心最直接最现实的利益问题，让改革发展成果更多更公平惠及全体人民。

各位代表，过去一年的成绩来之不易，令人振奋！这些成绩的取得，是党中央、国务院正确指引的结果，是省委、省政府和市委坚强领导的结果，是全市人民团结拼搏、社会各界共同努力的结果。在此，我代表市人民政府，向奋战在各行各业的全体劳动者，向人大代表、政协委员，向各民主党派、工商联和无党派人士、各人民团体，向离退休老同志，向中央和省驻宿单位以及人民解放军、武警、公安干警，向参与宿迁建设的海内外客商、广大援宿干部，以及所有关心支持宿迁发展的各级领导、各界人士，致以崇高的敬意和衷心的感谢！

在肯定成绩的同时，我们也清醒地看到，全市经济社会发展中还存在一些矛盾和问题：部分指标增幅有所回落，经济下行压力依然较大；产业总体仍处于价值链中低端，发展的质量和效益有待提高；实体经济体量较小，发展的基础和支撑还不稳固；生态环境还有不少短板，生态基础需要进一步夯实；发展不平衡不充分问题依然存在，城乡融合发展仍需继续发力；民生保障体系还不完善，公共服务与群众的期望还有一定差距；政府职能转变有待加快，新常态下组织经济工作能力亟需进一步提升，等等。对此，我们一定高度重视，切实采取措施，认真加以改进。

全力做好2018年政府重点工作

2018年是贯彻落实党的十九大精神的开局之年，是改革开放40周年，是决胜全面建成小康社会、实施“十三五”规划承上启下的关键一年，做好今年工作意义重大。党的十九大作出中国特色社会主义进入新时代的重大判断，描绘了中华民族伟大复兴的宏伟蓝图，随着党的十九大决策部署的深入实施，我国经济必将出现更多积极变化，我市经济社会发展也将迎来更好的机遇、享受更多的红利。同时，我国经济由高速增长阶段转向高质量发展阶段，正处在转变发展方式、优化经济结构、转换增长动力的攻关期，为宿迁实现“不走老路、加快发展”带来了难得的历史契机和更大的动力。我们坚信，有党中央、国务院的正确指引，有省委、省政府的坚强领导，有市委的有力带领，有市人大、市政协和社会各界的关心支持，有全市人民的共同努力，我们一定能够抢抓机遇、开拓创新，推动经济社会实现高质量发展。

今年政府工作的指导思想是：全面贯彻党的十九大精神，以习近平新时代中国特色社会主义思想为指导，按照习近平总书记视察江苏重要讲话精神和中央经济工作会议、省委十三届三次全会、市委五届五次全会部署安排，坚持稳中求进工作总基调，坚持新发展理念，坚持以人民为中心的发展思想，紧扣我国社会主要矛盾变化，按照高质量发展的要求，统筹推进“五位一体”总体布局、协调推进“四个全面”战略布局，深入推进“两聚一高”新实践，以供给侧结构性改革为主线，强化抓改革促发展、抓生态促转型、抓民生促和谐，突出抓重点、补短板、强弱项，扎实做好防范化解重大风险、精准脱贫、污染防治各项工作，更加彰显“江苏生态大公园”美好前景，奋力建设“强富美高”全面小康新宿迁。全市经济社会发展主要预期目标是：地区生产总值增长7%以上，一般公共预算收入增长2%左右，固定资产投资增长8.5%左右，社会消费品零售总额增长11%左右，全体居民人均可支配收入增长与经济增长同步，节能减排完成省下达的目标任务。

重点抓好以下七个方面的工作：

（一）坚持总量集聚与结构升级并举，全面提升产业能级，夯实高质量发展基础。落实高质量发展要求，加快推动产业转型升级，提高产业发展层次，努力构建现代产业体系。

聚焦结构调整，优化产业质态。围绕推动传统产业高端发展，对接“中国制造2025”江苏行动纲要，鼓励企业开展装备更新、技术升级、产品换代，推动传统产业提质升级。围绕推动新特产业跨越发展，抓好8个先进特色产业园建设，加快韩电电器、格力大松二期等项目建设，壮大智能家电规模；推进现代筑美家居、赛木科技等项目建设，做强绿色建材品牌；加快申龙项目建设进度，发展新能源汽车；积极培育数字经济、共享经济等新业态、新模式，大力发展激光、石墨烯等先导性产业。围绕推动现代服务业提速发展，加快推进项王故里三期、骆马湖影视基地、三台山—骆马湖山水绿廊、古黄河水上游等重点项目，启动恒大童世界建设和骆马湖—三台山国家级旅游度假区创建工作，进一步丰富旅游产品、延伸产业链条，推动一日游、观光游向多日游、休闲观光游转变；加快三台山国家森林公园健康颐养园等项目建设，打造健康养生产业新高地。

聚焦做优做强，提升产业层次。着眼提高招商质量，积极开展基金招商，探索邀约招商，加快引进一批龙头型、旗舰型项目，确保年内引进亿元以上项目超过210个；编制招商引资“正面清单”，提升入园项目质量。着眼打造千亿级产业，制定纺织服装、食品饮料等产业发展扶持政策，加快集聚产业配套项目和关键环节企业，推动传统制造业做大做强。强化责任落实，确保列市重大工业项目扎实推进。着眼做优载体，落实金融服务、公共服务设施等建设导则，进一步提高各类开发区承载服务能力。

聚焦科技支撑，促进产业升级。大力推进信息化和工业化深度融合，完成30个信息化改造提升重点项目，鼓励企业建设智能车间、智能工厂；支持企业开展个性化定制，年内定制经济销售总额超5亿元；完善电商公共服务体系，培育壮大8个省级电商示范基地。加强宿迁（上海）协同创新基地建设，鼓励企业建设离岸研发机构，支持企业与“双一流”大学共建企业研究院。创新终端成果转化机制，充分发挥技术交易服务中心作用，让更多科技成果落地转化。加快创建全国质量强市示范城市。扎实开展柔性引才，积极引进领军人才、名校优生、技能人才等各类人才。

（二）坚持以提高质量和效益为重点，营造良好发展环境，稳定实体经济运行。全面深化供给侧结构性改革，加强运行调度，强化要素支持，优化服务供给，推动实体经济持续健康发展。

扎实抓好“三去一降一补”。去产能方面，坚持用市场化、法治化手段，进一步化解过剩产能、淘汰落后产能。去库存方面，坚持“房子是用来住的、不是用来炒的”，深入落实“七优”政策，推动房地产业持续平稳健康发展。去杠杆方面，支持企业利用中期票据、股权融资等手段，进一步提高直接融资比重，争取全年新增直接融资220亿元。打好防范重大风险攻坚战，加强对企业担保圈、非法集资等风险的分类处置，确保不发生区域性、系统性金融风险。加强政府债务管理，完善全口径债务管理化解机制，逐步化解融资平台债务。降成本方面，在降低税负成本、要素成本上协同发力，全面清理规范涉企收费。补短板方面，积极推进重大项目、中心城市重点基础设施项目和为民办实事项目，全力补好重点产业、重大基础设施、生态环保、民生等领域短板。

深入实施“四大行动”。引导兼并重组，支持优质企业与国内外上市公司、行业龙头企业合作，实现品牌价值、营销渠道、工艺技术和装备水平的优势互补。鼓励技术改造，引导企业“设备换芯”“生产换线”“机器换人”，实现提档升级。推动股改上市，支持更多企业挂牌上市。促进规模发展，引导企业提升创新能力、拓宽供需渠道、升级产品服务。

切实强化要素保障。优化用能供给，推广天然气大用户合同交易，扩大直购电规模，改进企业电费缴纳及减产停产期间电费计费方式。优化金融供给，引导银行机构推广信用贷款，降低企业信贷成本；加大创投、风投、天使基金等引进力度，为企业提供多元化金融服务。优化人力资源供给，鼓励外出务工人员返乡就业，探索与中西部地区共建多种形式的劳动力基地，吸引更多劳动力资源向宿迁集聚。优化物流服务供给，加快传化公路港、江苏通湖物流园等重点项目，以及公共物流场站和信息平台建设，打造区域商品集散地。

全力优化营商环境。完善营商环境评价指标体系，发布区域评价指数，为营商环境评价提供更好依据。加大考核力度，综合运用正面激励、负面惩处等措施，推动各地强化营商环境建设。营造良好法治环境，加强财产权和知识产权保护，激发和保护企业家精神。优化帮办服务，深入开展“三服务三促进”活动，帮助化解难题，增强发展信心，助力企业健康稳定发展。

（三）坚持农业农村优先发展，深入实施乡村振兴战略，推动“三农”工作再上新台阶。按照“产业兴旺、生态宜居、乡风文明、治理有效、生活富裕”的总要求，加快推进农业农村现代化。

打造品牌农业。推进农业工业化生产，深入实施农业生产机器换人、绿色环保装备与技术示范应用“两大工程”，着力提升农机装备水平。推进农业规模化生产，引导土地向大户、农场和龙头企业集中，全年建成2000亩左右成方连片高效农业基地25个。新建高标准农田40万亩，加快冷链物流体系和冷链加工体系建设，提高农业生产服务设施配套水平。落实粮食安全责任制，确保区域粮食安全。大力发展绿色规模种养，加快建设生态健康渔业示范区，打响生态农业品牌。推进农业标准化生产，加快标准体系建设与推广，年内农业标准化普及率达40%，促进农业由增产量向提质量转变。

培育新型经营主体。加大农业产业化龙头企业引培力度，新培育市级以上龙头企业10个；加强农业加工流通企业与电商企业的引进和培育，推进“互联网+现代农业”，打造贯穿一二三产的产业链条。大力发展农村电子商务，确保“一村一品一店”建成率达70%以上。积极培育职业农民、专业大户、农民合作社等经营主体，新建家庭农场集群及综合服务中心10个以上，向农业输入现代生产要素和现代经营模式，真正让农民成为体面的职业，让农业成为有效益、有奔头的产业。

建设美丽乡村。围绕好产业、好设施、好机制、好环境、好生活“五好标准”，加快建设5个特色田园乡村。扎实推进8个生态经济示范镇建设，积极探索生态保护、产业转型、改革创新、富民增收的示范之路。提高农村生产生活配套设施等级，加快“四好农村路”建设，完成1200公里安全生命防护工程；实施“点亮乡村”工程，让群众出行更便利、更亮堂；推动等级公厕进镇入村，努力让农村居民享有与城市居民均等化的公共服务。开展和谐乡村等创建活动，打造乡风文明、有礼有序的魅力乡村。

（四）坚持强功能塑个性增活力，不断增强城市能级，提升中心城市首位度。按照“生态、精致、时尚，充满人文关怀”建设定位，努力建设人民满意城市。

增强城市活力。以中央活动区建设为引领，着力繁荣以宝龙、金鹰、水韵城为主的核心商圈，改造提升新盛街片区、宝龙二十四街、楚街等商业街区，充实和丰富霸王举鼎广场、仁恒里文创街区商业业态，推进西楚活力街区、苏州风情街、万达广场等项目建设，加快培育城市活力源。创新消费奖补机制，吸引周边人群来宿消费，繁荣城市经济；积极支持开展各类文化艺术演出活动，不断增强中心城市辐射力和集聚力。

优化城市功能。深入推进厕所、农贸市场和垃圾“三场革命”，全年新建14座“街坊公舍”；中心城区合理布局1-2个农产品批发市场，新建10个标准化农贸市场；中心城市建成区垃圾分类设施覆盖率达40%。按照“公园广场+”理念，推进小型体育设施、文化设施、游乐设施、活力街区、便民摊点“五小建设”，推广城市智能家具，让市民更多走出家门、共享美好生活。加快设置小区二代集中晾晒区、非机动车停放区、充电桩等便民设施，进一步提升物业管理水平。继续实施道路通达工程，新建5条主干路及40条支路，改造34条道路，着力缓解交通拥堵。推动互联网、云计算与城市治理与服务融合发展，探索推进交通、能源、供水等基础设施数据化，打造城市智慧大脑。

精塑城市特色。围绕建设林荫、彩色、花园、海绵“四个城市”，全面推行“333”工作法，年内新（改）建30个街头绿地、小型游园，打造30条落英落叶景观路，完成10家单位庭院开放式改造。实施28条道路路灯、景观灯改造工程，做好楼体、水体、树体、桥体“四体”亮化，进一步扮靓城市夜景。实施街区更新计划和“趣城”计划，启动10条街区创新性改造，更新城市灰空间。推广使用青砖青瓦、红砖红瓦等宿迁记忆中的建筑材料，打造独特的建筑元素和符号。推进骆马湖宿迁大三角控制区退田还湖生态工程，促进宿迁由湖滨城市向亲水城市转变。

提升城市文明。实施“文明20条”拓展延伸工程，出台宿迁城市文明行为“正面清单”，形成宿迁文明建设新亮点。推动文明建设由市区向县城拓展、向农村延伸，形成人人文明、处处文明的良好风尚。发挥政府与社会协同作用，建立健全诉求表达、矛盾调处、权益保障等机制，缓冲社会矛盾、纾解社会焦虑，培育理性平和、向善向上的社会心态。

提高城市通达力。加快综合客运枢纽、上海路、迎宾大道建设，徐宿淮盐高铁开展全线铺轨，合宿新高铁争取年内开工，启动宿连高速和大兴、泗洪通用机场等前期工作，完成宿连航道初步设计。推进S330宿迁段、S329沭阳段等国省干线公路建设，S330跨溧河洼特大桥及接线工程年内建成通车。抢抓省探索高铁自主规划建设运营模式的契机，加强相关高铁项目研究、策划、对接，力争有新项目进盘子。

（五）坚持源头治理和生态修复并重，持续优化生态环境，提高经济社会发展承载能力。深入践行“两山”理论，推进生态经济示范区和“江苏生态大公园”建设，坚决打好污染防治攻坚战，让碧水蓝天成为常态。

加强生态保护与治理。深入开展“263”专项行动，强化大气污染治理，推进“清风廊道”建设，完成国家、省下达的空气质量提升任务，全力打赢蓝天保卫战。严格落实“水十条”，系统推进民便河等17条黑臭水体整治，启动农村河道清流及连通工程。全面落实“土十条”，完成农用地土壤污染详查，建立污染地块名录和开发利用“负面清单”；加强农业面源污染防治，推动农药化肥减量，推进土地盐碱化整治，着力解决土地连作障碍问题。开展生态修复，巩固“两湖”禁采成果，确保完成退渔还湖任务；加大自然湿地保护力度，年内创成骆马湖省级湿地公园。

提高生态环境容量。围绕提升污染物收集能力，实施城市雨污分流改造、城乡生活垃圾分类收集、尾水导流工程，推进重点区域控源截污和污水管网建设。围绕提升污染物处置能力，加强建筑垃圾资源化利用，加快生活垃圾焚烧、餐厨废弃物处理等终端设施建设，完成河西污水处理厂二期工程，乡镇污水处理设施实现全覆盖，40%以上行政村实现污水集中处理；健全农业废弃物处置奖励政策，秸秆综合利用率提高到98%。围绕提升清洁能源供应能力，积极推进“煤改气”“煤改电”；加快天然气热电联产项目建设，年内建成运行；做好城市应急储备气源站建设，力争年内一期工程投入使用；推动天然气管网向乡镇延伸，让更多群众用上清洁安全的能源。围绕提升碳汇能力，以创建国家森林城市为抓手，加快100万亩生态经济林建设，更新改造杨树26.16万亩。

优化生态建设机制。坚持用最严格的制度、最严密的法治保护生态环境，健全环境保护责任体系以及生态环境损害赔偿、生态补偿制度，严肃查处破坏环境行为。排出招商引资和项目建设环保准入“负面清单”，从源头上减少污染。推进环保信用评价，进一步扩大参评企业范围。完善绿色交易平台，全面推行碳排放交易、排污权交易，探索建立市场化的生态补偿机制。支持金融机构发展碳排放权、排污权等质押融资产品，引导企业绿色发展。

（六）牢牢把握“减权去利便利化”，坚定不移全面深化改革，进一步释放市场和社会主体活力。坚持把发挥好市场、政府“两个作用”作为核心，把激发市场和社会活力作为主要任务，加大改革攻坚力度，更大程度释放制度生产力。

深入推进经济体制改革。着眼更加深入的“放”，全面实行准入前国民待遇加负面清单管理制度，放宽市场准入；推进“以信用简化审批”改革，进一步精简审批事项；深化“多证合一”和“证照分离”改革，推进“照后减证”，破解“准入不准营”问题；深化资格资质改革，着力打造“2+2”改革模式，争创国家职业资格改革试点；深化工业类产品生产许可制度改革，扩大“先证后核”范围，探索建立产品质量管理的“宿迁模式”。着眼更高水平的“管”，深化综合行政执法改革，完善“五位一体”综合监管体系，进一步提升事中事后监管实效。着眼更高质量的“服”，加快完善政务服务“一张网”，推进“不见面”审批标准化、规范化，固化“3550”改革成果；加快推进数据信息共享共用，变“群众奔波”为“信息跑腿”；加强乡镇（街道）、村（社区）代办帮办服务点建设，切实打通政务服务“最后一公里”。

深入推进农业农村改革。不断深化农村土地制度、集体产权制度等改革，建设城乡统一的人才、土地、科技、资本等要素市场，推动城乡融合发展。落实农村土地“三权分置”，深化土地增减挂钩改革，按市场导向成立土地资产经营管理公司，让更多农民变为股民。推动农村“三资”管理改革，开展涉农资金、扶贫资金形成的资金资产折股量化试点，继续做好“两权”抵押贷款试点。稳步推进农村不动产统一登记，建立自愿有偿退出农村宅基地机制，释放土地活力。

深入推进社会事业改革。深化教育改革，推进中心城区基础教育阳光招生，提高义务教育招生工作公信力；推进职业教育改革，加快职业教育服务经济发展试验区建设；制定促进民办教育发展意见和分类管理办法，推进民办学校规范发展、特色发展。深化医药卫生体制改革，鼓励社会资本新建高等级专科医院，探索二级以上社会办医机构实施药品零差率销售；完善医保结算方式，引导群众合理有序就医；健全分级诊疗制度，完善家庭医生签约服务，进一步提升医疗服务水平。统筹做好其他各项改革。

（七）坚持以人民为中心的发展思想，加大民生保障和改善力度，不断增强群众获得感和幸福感。聚焦“医教水住行”“法访文保收”，扎实办好民生实事，着力补齐民生短板，优化公共服务供给，更好地满足人民群众对美好生活的向往。

大力发展社会事业。积极推动就业创业，开展就业创业“春风行动”，实现城镇新增就业5万人；深入实施“五小创业”，引导更多群体投身创业、带动就业。推动教育事业均衡发展，优化中心城区基础教育布局，确保青海湖路学校、钟吾初中整体改造项目建成投用，启动11所中小学校和13所幼儿园建设，落实义务教育学校免试就近入学政策，着力化解“择校热”“大班额”等问题；加大教师引培力度，建强教师队伍；深入实施义务教育改薄工程、“暖心工程”。加快医疗卫生事业发展，支持市人民医院创建三甲医院；完成新一轮村居卫生室提档升级，实施乡镇医院能力提升工程，支持医疗人才引进和培养。繁荣文体事业，深入开展“文化四季”惠民演出等活动，用群众喜闻乐见的形式加强社会主义核心价值观教育；完善“十分钟健身圈”功能，办好“1+X+1”系列赛事，切实增强时尚体育城市影响力。

提升社会保障水平。深入实施全民参保计划，城乡居民基本医疗保险覆盖率达98%以上、低收入农户城乡居民基本养老和医保参保率100%。落实低保、农村“五保”、城市“三无”、城乡孤弃儿童保障标准增长机制，全力做好特困群体基本生活保障。加快居家、社区、机构养老服务一体发展，促进医养融合，进一步优化养老服务供给。加快建立多主体供给、多渠道保障、租购并举的住房制度，满足群众多层次的住房需求。

打好精准扶贫攻坚战。用足用好国家扶贫改革试验区政策，突出“三大片区”整体帮扶，加快推进15个试点项目建设，不断提升扶贫开发的针对性和实效性。扎实开展“三进三帮”“挂村包户”活动，深入落实扶贫“六项机制”，全面推行“阳光扶贫”，加大产业扶贫、电商扶贫、股权扶贫、保险扶贫力度，年内完成10万低收入人口和40个经济薄弱村脱贫，确保全面小康路上“一个不少、一户不落”。

加强和创新社会治理。完善多元化治理，支持公益慈善、社区服务、乡贤协会等各类社会组织发展，发挥好社会组织和广大乡贤在社会治理中的重要作用。开展信用体系建设“四个试点”，构建信用承诺+契约管理的工作机制，提升信用约束力。突出标准化治理，实施新一轮城乡技防建设，打造社会治安防控体系升级版。推进专业化治理，发展社会专业服务机构，加强社会工作专业人才队伍和志愿者队伍建设，健全网格化社会治理机制。深入开展平安宿迁建设，常态化打击“盗抢骗”“食药环”等违法犯罪活动。推进法治信访和阳光信访建设，营造和谐稳定的社会环境。

全面提升政府自身建设水平

新时代召唤新作为，新担当不负新使命。我们将把学习宣传贯彻党的十九大精神作为首要政治任务，深刻领会习近平新时代中国特色社会主义思想，牢固树立“四个意识”，不断提高政治站位和政治自觉，不忘初心，牢记使命，全面加强政府自身建设，努力开创各项工作新局面。

坚持系统思维，整体谋划科学施政。坚决贯彻市委决策部署，确保各项任务落地见效。工作目标上，把宿迁发展放到国内外大形势、大格局中去思考、研究和把握，明晰方向和重点，切实提高工作的前瞻性、系统性、科学性。工作方法上，注重动力机制和反馈机制的协调，进一步提升解决复杂问题的能力。工作推进上，强化综合施策，坚持“十个指头弹钢琴”，保障各项工作衔接有序、相互促进。工作效果上，注重速度与质量的统一，兼顾当前需要与长远发展，努力实现“一加一大于二”。

增强法治理念，规范权力依法行政。落实市政府常务会集体学法、法律顾问等制度，自觉运用法治思维和法治方式推动工作，切实提高依法行政水平。遵循“三重一大”事项集体决策、民意征集、专家咨询论证等制度，健全重大决策合法性、风险性和廉洁性审查评估机制，确保依法决策、科学决策。加快推进政务公开向深层次延伸，加强信息发布和政策解读，及时回应社会关切。

强化精细管理，狠抓落实高效勤政。坚持以项目化思路推进工作，把工作细化为一件件看得见、摸得着的项目，明确每个环节的责任清单和责任边界，制定工作路线图和时间表，使目标更加清晰、任务更加具体。把项目化考核贯穿工作全过程，加强过程管理和结果问责，推动工作有力有序开展。大兴调查研究之风，深入基层一线，察实情、出实招、见实效。

严守纪律规矩，保持本色廉洁从政。严格遵守中央八项规定《实施细则》以及省委、市委《具体办法》，持续不懈地纠正“四风”，锲而不舍地抓好作风建设。深入落实党风廉政建设责任制，进一步健全廉政建设与业务工作融合机制。强化执纪问责，始终保持反腐败斗争高压态势，严查发生在群众身边的不正之风和腐败行为，全力打造风清气正的政府形象。

各位代表！宿迁发展的历史使命，鼓舞我们砥砺奋进；全市人民的信任期盼，鞭策我们勇往直前。让我们紧密团结在以习近平同志为核心的党中央周围，以习近平新时代中国特色社会主义思想为指导，在省委、省政府和市委的坚强领导下，在市人大、市政协和社会各界的监督支持下，同心同德，开拓创新，为决胜“强富美高”全面小康新宿迁、谱写好伟大中国梦的宿迁篇章而努力奋斗！
